# Cross-Classification Analysis of Food Products Based on Nutritional Quality and Degree of Processing

**DOI:** 10.3390/nu15143117

**Published:** 2023-07-12

**Authors:** Sandra Abreu, Margarida Liz Martins

**Affiliations:** 1 School of Life Sciences and Environment, University of Trás-os-Montes, and Alto Douro (UTAD), 5000-801 Vila Real, Portugal; 2 Research Centre in Physical Activity, Health, and Leisure (CIAFEL), Faculty of Sport, University of Porto, 4200-450 Porto, Portugal; 3 Laboratory for Integrative and Translational Research in Population Health, 4050-600 Porto, Portugal; 4 Polytechnic Institute of Coimbra, Coimbra Health School (ESTeSC), 3045-093 Coimbra, Portugal; margarida.liz@estesc.ipc.pt; 5 GreenUPorto—Sustainable Agrifood Production Research Centre, 4200-465 Vairão, Portugal; 6 Centre for the Research and Technology of Agro-Environmental and Biological Sciences (CITAB), 5000-801 Vila Real, Portugal

**Keywords:** food classification, food label, nutritive value, ultra-processed foods

## Abstract

This study aims to compare the classification of foods available in the Portuguese market using Nutri-Score and NOVA classifications and to analyse their ability to discriminate the fat, saturated fat, sugar, and salt content of foods. A sample of 2682 food products was collected. The nutritional quality of foods was established using the Nutri-Score, classifying them into five categories (from A to E). The NOVA classification was used to classify foods according to the degree of food processing into unprocessed/minimally processed foods, processed culinary ingredients, processed foods, and ultra-processed foods (UPF). The nutritional content of food products was classified using a Multiple Traffic Light label system. It was observed that 73.7% of UPF were classified as Nutri-Score C, D, and E, 10.1% as Nutri-Score A, and 16.2% as Nutri-Score B. Nutri-Score was positively correlated with NOVA classification (ρ = 0.140, *p* < 0.001) and with the Multiple Traffic Lights system (ρ_Total Fat_ = 0.572, ρ_Saturated Fat_ = 0.668, ρ_Sugar_ = 0.215, ρ_Salt_ = 0.321, *p* < 0.001). NOVA classification negatively correlated with the Multiple Traffic Lights system for total fat (ρ = −0.064, *p* < 0.001). Our findings indicate the presence of many UPFs in all Nutri-Score categories. Since food processing and nutritional quality are complementary, both should be considered in labelling.

## 1. Introduction

In 2019, 14.1% of global deaths—equivalent to approximately 8 million—were associated with poor diet, which is highly correlated to developing a range of chronic diseases, including obesity, diabetes, cancer, and cardiovascular diseases [1,2,3]. Diets high in sodium and red and processed meat or low in whole grains, pulses, fruit, nuts and seeds, and vegetables are more likely to contribute to increased mortality and the loss of healthy life years [4]. Furthermore, according to the Global Burden of Diseases, Injuries, and Risk Factors Study (GBD) 2019, risk exposure to unhealthy foods such as sugar-sweetened beverages increased from 1990 to 2019 [1]. Thus, there is a need to assist consumers in making informed and healthy food choices, potentially leading to a healthier diet.

In this context, food and nutrition policies have been set up by governments to modify the food environment and improve the nutritional quality of food and consumers’ food choices. Nutrition labelling, particularly front-of-pack labelling (FOPL), has been used as a cost-effective policy tool to promote healthy diets and prevent obesity and diet-related non-communicable diseases [5,6]. FOPL was used for the first time in the late 1980s in Sweden and today is widely present in more than 30 countries [7,8]. Several FOLPs have been developed with different formats (e.g., stars, traffic lights, stop signs) using either interpretative (i.e., providing guidance on the relative healthfulness of a food product) or non-interpretative (i.e., not providing advice or direction on the overall nutritional value of the food) systems [5,8,9]. In Portugal, various FOPL schemes are present in packaged, manufactured, or processed foods, such as the Multiple Traffic Lights, the Reference Intake format, and the Nutri-Score [9]. The National Program for the Promotion of Healthy Eating (PNPAS) proposed a decoder for reading labels [10] to assist consumers in making more informed and healthier choices to classify each product. This decoder was based on the United Kingdom’s Multiple Traffic Light label and classified food products according to their content of total fat, saturated fat, sugar, and salt [11].

The front-of-pack label Nutri-Score is widely present in food products and is a system based on a five-colour and letter nutritional scale (from green/A to red/E) that considers the overall nutritional value of foods. A growing body of evidence suggests that Nutri-Score improves the overall quality of consumers’ food choices [12,13,14]. A cross-sectional study with 1059 Portuguese adults found that Nutri-Score compared to other FOPL systems (Health Star Rating, Multiple Traffic Lights, Reference Intakes or Warning symbol), was the most efficient in informing consumers about the nutritional quality of foods and in assisting them to make healthier options [9]. Furthermore, recent studies show that consuming foods with an unfavorable Nutri-Score rating was associated with a higher risk of non-communicable diseases (like cancer) and mortality [15,16,17].

Although the Nutri-Score helps consumers understand the overall nutritional value of foods until now, the scores did not account for the presence of additives, such as flavoring agents, sweeteners, and the degree of food processing. In the last few years, evidence has emerged on the relationship between the degree of food processing and the risk of developing non-communicable diseases [18,19,20,21]. Food processing may affect health through various mechanisms, including altered inflammation, satiety and glycemic responses, and gut microbiota composition and function [22]. Considering the growing interest in the effect of food processing on health, Monteiro et al. [23] proposed the NOVA food classification system as a simple method to classify foods according to the nature, extent, and purpose of industrial processing in four categories: (i) minimally processed foods; (ii) processed culinary ingredients; (iii) processed foods; (iv) and ultra-processed foods (UPF). NOVA classification has been widely used in epidemiological studies to classify foods according to their processing degree and relationship with the risk of developing non-communicable diseases [19,20,24].

To date, according to our knowledge, few studies have investigated the agreement between food processing extent and the FOPL system. A study in Spanish markets found that all Nutri-Score categories include at least 26% of UPF, suggesting that FOPL should also have information on the degree of food processing [25].

Thus, the present study aims to compare the classification of a sample of foods available in the Portuguese market using Nutri-Score and NOVA classifications. Additionally, we analyze the ability of Nutri-Score and NOVA classifications to discriminate the content of food in fat, saturated fat, sugar, and salt using a multiple traffic lights label system.

## 2. Materials and Methods

### 2.1. Data Collection

A cross-sectional study was carried out. A random sample of 2682 food products was collected and analyzed between December 2021 and December 2022 from national hypermarkets and supermarkets. Data collection was performed either presential or through market websites. For each food product evaluated, a photograph or screenshot (when the collection was carried out online) of the label was collected to obtain the list of ingredients and nutritional information.

### 2.2. Food Classification

All foods evaluated were classified into 12 groups and 27 subgroups, considering the classification of FoodEx 2 [26] (Appendix A, Table A1).

### 2.3. Nutri-Score

The nutritional quality of food was established using Nutri-Score. The Food Standards Agency Nutrient Profiling System score (FSAm-NPS score) was calculated using the nutritional information in packaging to determine Nutri-Score categories. FSAm-NPS score is based on the nutritional composition of 100 g of food (or 100 mL of beverage) and ranges from +40 (least healthy) to −15 (most healthy). Energy, sugar, saturated fat, and sodium score negative points, while fruit and vegetables, nuts and pulses, protein and fibre score positive points. The total sum of the score is divided into five groups: (i) group A includes scores between −15 and −1 for solid foods or water; (ii) group B includes scores between 0 and 2 for solids food and scores between −15 and 1 for beverages; (iii) group C includes scores between 3 and 10 for solid foods and scores between 2 and 5 for beverages; (iv) group D includes scores between 11 and 18 for solid foods, and scores between 6 and 9 for beverages; (v) and group E includes scores between 19 and 40 for solid foods and scores between 10 and 40 for beverages [27]. As information on dietary fibre and percentage of fruit and vegetables, nuts, and pulses are not mandatory in food labelling, we used previously described procedures [28,29]. For the food subgroup in which the mean amount of dietary fibre is described as lower than 0.9 g we considered 0 value (Meat, seafood, Cheese and Curd, Vegetal oils, Olive oils, Dairy cream, Milk, Soft drinks). For other products, dietary fibre was determined using the following equation: energy (kJ) = total carbohydrates (g) × 17 + total protein (g) × 17 + total fat (g) × 38 + alcohol (g) × 29 + total dietary fibre (g) × 8. All values were checked and when they exceeded two standard deviations of its subgroup, the quantity of dietary fibre was estimated using similar products. For the percentage of fruit and vegetables, nuts, and pulses, we considered a standard value according to legislation or matching with similar products.

### 2.4. NOVA Classification

Food products were classified according to the NOVA classification [23,30], considering the degree of food processing. NOVA establishes four groups: (i) Unprocessed or minimally processed foods (NOVA 1) include edible parts of plants (seeds, fruits, leaves, stems, roots) or of animals (muscle, offal, eggs, milk), and also fungi, algae and water, after separation from nature, as well as, natural foods altered by processes that include removal of inedible or unwanted parts, and drying, crushing, grinding, fractioning, filtering, roasting, boiling, non-alcoholic fermentation, pasteurization, refrigeration, chilling, freezing, placing in containers and vacuum-packaging; (ii) Processed culinary ingredients (NOVA 2) comprises substances derived from NOVA 1 or nature by processes that include pressing, refining, grinding, milling and drying. Oils, butter, sugar, and salt are examples of processed culinary ingredients; (iii) Processed foods (NOVA 3) usually result from combining substances (oil, sugar, salt) from NOVA 2 to NOVA 1 foods and are recognizable as modified versions of NOVA 1 foods. Processing includes preservation and cooking methods and non-alcoholic fermentation to increase food shelf life and optimize sensory characteristics. Most processed foods have two or three ingredients; (iv) UPF (NOVA 4) are formulations that result from substances derived from foods and additives, with little if any intact NOVA 1 food products. These formulations include processed food ingredients, such as sugar, oil or salt and other sources of energy and nutrients not usually used in culinary preparations, some of them extracted from foods (casein, lactose, whey, gluten), others derived from food processing constituents (such as hydrogenated or interesterified oils, hydrolyzed proteins, soya protein isolate, maltodextrin, invert sugar and high-fructose corn syrup) and additives (such as preservatives, antioxidants and stabilizers, flavors, flavor enhancers, non-sugar sweeteners; and processing aids). Ultra-processing includes hydrogenation and hydrolyzation, extrusion, molding and pre-processing for frying to create branded, convenient, attractive, palatable and highly profitable food products [23].

### 2.5. Multiple Traffic Lights System

We used the decoder for reading labels proposed by the PNPAS [10] and based on the Multiple Traffic Lights system to classify each product according to its total fat, saturated fat, sugars, and salt content. Thus, the amount of total fat, saturated fat, salt, and sugar per 100 g/100 mL was classified as high (red), medium (yellow) or low (green) according to the information in Table 1.

### 2.6. Statistical Analysis

Statistical analyses were performed using the IBM Statistical Package for the Social Sciences for Windows^®^ (Version 27.0. IBM Corp., Armonk, NY, USA). Descriptive data were expressed as absolute and relative frequencies, and continuous variables as median (percentiles 25 and 75). The correlation between the three systems to classify food products (Nutri-Score, NOVA, and Multiple Traffic Lights) was analysed by categorical principal component analysis (CATPCA) and Spearman’s correlation coefficients. A *p*-value < 0.05 was regarded as significant.

## 3. Results

Of all food products analyzed in this study and according to the Nutri-Score, it was observed that 71.6% were classified in categories C (24%), D (26%) and E (21.6%). Considering the NOVA classification, the group with the highest frequency (84.8%) was UPF (NOVA 4).

**Figure 1:**
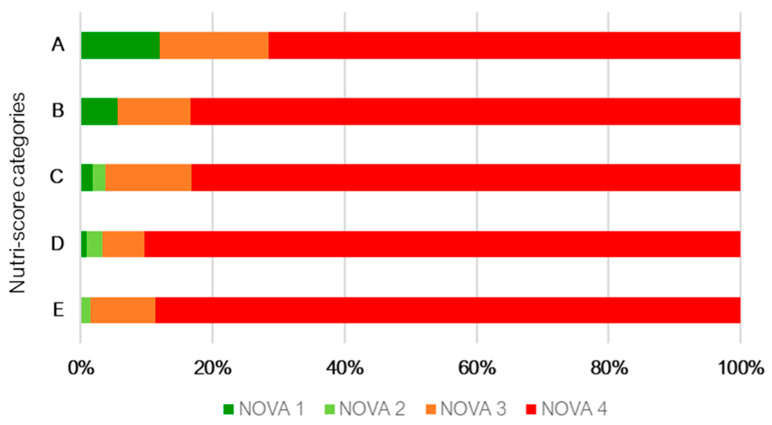
Distribution of NOVA classification (NOVA 1: Unprocessed or minimally processed foods; NOVA 2: Processed culinary ingredients; NOVA 3: Processed foods; NOVA 4: Ultra-processed foods) within Nutri-Score categories (A; B; C; D; E).

Figure 1 presents the cross-distribution between Nutri-Score and NOVA classification. Regardless of the Nutri-Score category, most products are classified as ultra-processed products (NOVA 4), representing 71.5% to 90.3% of the products analyzed.

Considering NOVA 4 food products, 73.7% were classified as Nutri-Score C, D and E, 10.1% as Nutri-Score A and 16.2% as Nutri-Score B.

Table 2 presents the distribution of food products according to Nutri-Score and NOVA classifications. According to Nutri-Score, it was observed that 90.4% of pulses, 93.0% of milk, 83% of milk and dairy products substitutes, 80% of potatoes and other tubers, 80% of rice and other grains, 78.6% of vegetables and 74.6% of yoghurt and other fermented milk were classified as A or B (Table 2). On the other hand, 100% of dairy cream, butter, and other fats, 95.5% of vegetable oils, 89.1% of cakes, 83.2% of meat, 80.9% of nectars, 88.8% of margarine and minarines, 76.3% of biscuits and commercial cookies, and 72.2% of sweets were classified as D or E (Table 2). When analysing food products according to the NOVA classification, it was observed that all bread and toasts, margarines and minarines, other fats, cakes, biscuits, commercial cookies, stuffed and fried patties and pizzas, nectars, soft drinks, and other products were graded as NOVA 4. Additionally, almost sweets (98.7%), meat substitutes (96.6%), breakfast cereals and cereal bars (95.9%), meat (95.5%), milk (95.3%), dairy cream (94.1%), and yogurt and other fermented milk (91.4%) were also classified as NOVA 4 (Table 2). NOVA 1 classification was present in higher prevalence in nuts and seeds (63.3%), processed fruit (42.4%), natural fruit juices and 100% juices (39.1%), vegetables (25.8%) and rice and other grains (20%) (Table 2).

Considering all food products classified as NOVA 4, it was observed that dairy products were the food group that most contributed to the percentage of foods classified as Nutri-Score A (30.3%) and B (40.4%). Moreover, yogurt and other fermented milk represented the food sub-group with the highest contribution for these categories (23.2% classified with Nutri-Score A and 31.2% with Nutri-Score B). Besides this, it was observed that cereals, derivatives, and tubers had a high contribution to the percentage of foods classified with Nutri-Score A (29.8%), mainly resulting from bread and toast (15.8%). Fruits, vegetables, and pulses also contributed significantly to the percentage of foods classified with Nutri-Score A (10.5%). Meat substitutes, sweets, cakes and cookies, and milk and dairy products substitutes represented 10.5%, 3.9% and 7.0% of food products classified as NOVA 4 and Nutri-Score A. Additionally, meat, seafood and eggs, milk and dairy substitutes, snacks, pretzels and pizzas represented 14.4%, 8.4% and 8.1% of food products classified as NOVA 4 and Nutri-Score B.

**Figure 2:**
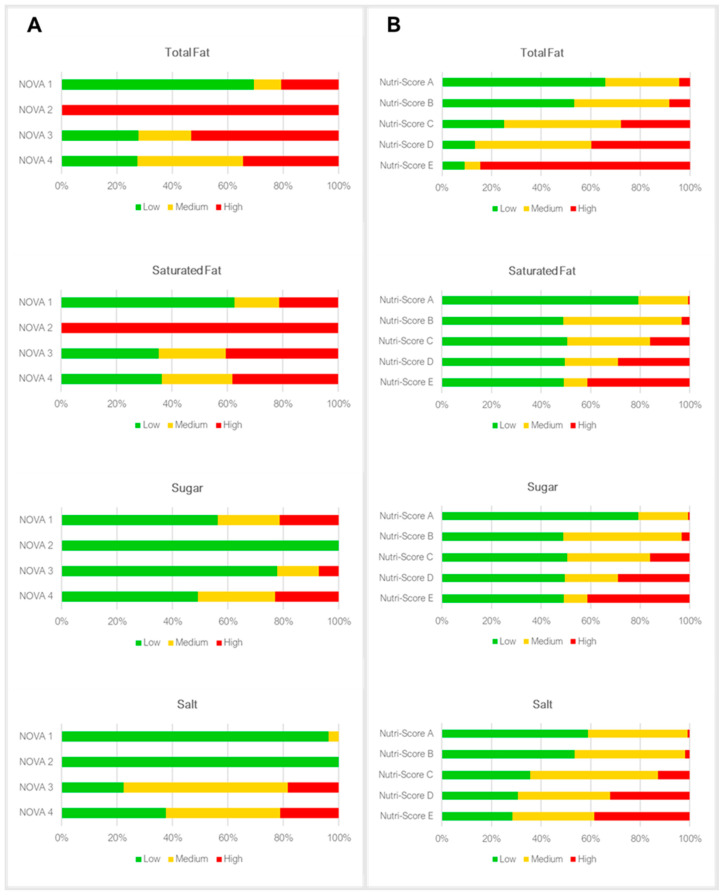
Distribution of Multiple Traffic Lights system for total and saturated fat, sugar and salt (Low: green; Medium: Yellow; High: Red) within NOVA classification (**A**) and Nutri-Score categories (**B**).

Figure 2 shows the distribution of high, medium, and low total fat, saturated fat, sugar and salt content for NOVA classification and Nutri-Score categories. All products in the NOVA 2 group have high total fat and saturated fat content. A higher proportion of products with high sugar content was found in NOVA 4 group (22.9%). NOVA 3 and 4 presented a higher proportion of products with high amounts of salt (18.0% and 21.2%, respectively). Overall, products with Nutri-Score grades C, D and E have a higher proportion of foods/beverages with high total fat, saturated fat, sugar and salt content.

**Figure 3:**
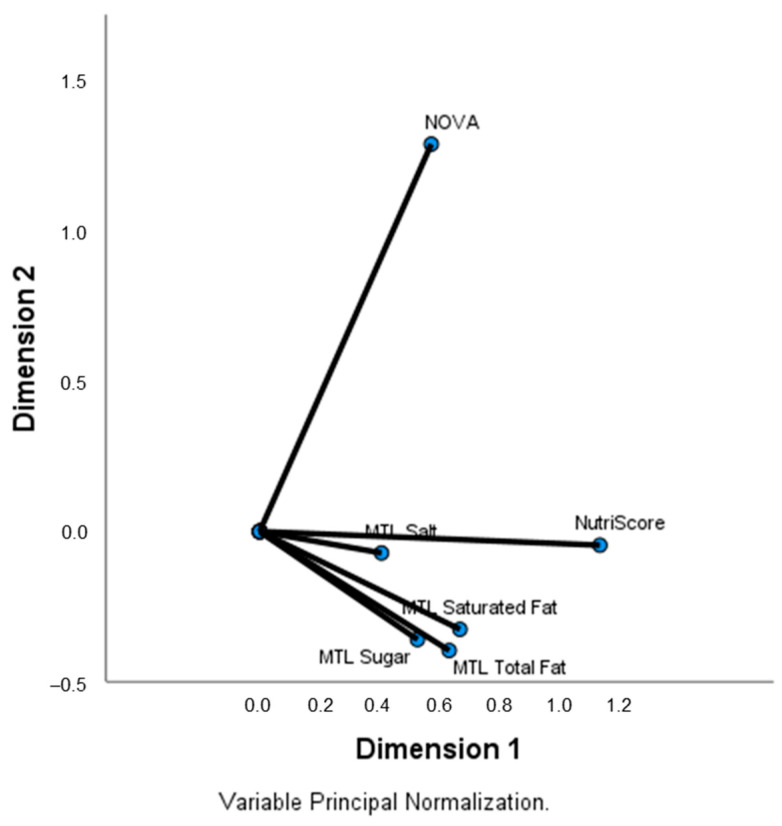
Categorical principal component analysis of variables (CATPCA) for Multiple Traffic Lights system (MTL), NOVA classification and Nutri-Score categories.

Figure 3 shows a clusterization between Nutri-Score and the Multiple Traffic Lights system. Nutri-Score was positively correlated with NOVA classification (ρ = 0.140, *p* < 0.001) and Multiple Traffic Lights system (ρ_Total Fat_ = 0.572, ρ_Saturated Fat_ = 0.668, ρ_Sugar_ = 0.215, ρ_Salt_ = 0.321, *p* < 0.001 for all). NOVA classification negatively correlates with the Multiple Traffic Lights system for total fat (ρ_Total Fat_ = −0.064, *p* < 0.001). It positively correlates with the Multiple Traffic Lights system for total sugar (ρ_Sugar_ = 0.184, *p* < 0.001) and salt (ρ_Sugar_ = 0.082, *p* < 0.001).

## 4. Discussion

The present study shows that almost three-quarters of UPF is classified as Nutri-Score C, D and E, representing foods with medium to low nutritional quality. Furthermore, UPF are present in all Nutri-Score categories, from 71.5% in Nutri-Score A to 90.3% in Nutri-Score D. It is usually described that UPF is mostly high in energy, added sugar, fats, and sodium and low in fibre and micronutrients [31], therefore it was expected that there would be a lower proportion of NOVA 4 foods in the highest nutritional quality Nutri-Score categories (A and B). In line with our findings, a study carried out in the Spanish market found that 75.5% of UPFs were rated as Nutri-Score C, D, and E. Furthermore, UPF is found in all Nutri-Score categories ranging from approximately 26% in Nutri-Score A to 84% in Nutri-score E [25]. Although Nutri-Score, as a nutrient profiling system, can discriminate the nutritional quality of foods and beverages, it cannot identify highly processed foods.

It is known that over-processing, characterized by either high manipulation of food through multiple processes or the addition of artificial ingredients, can lead to the development of compounds in food that are harmful to health, such as acrylamide, acrolein, endocrine-disrupting chemicals and phthalates [32,33,34]. Likewise, over-processing can deteriorate the food matrix, impairing or modifying the bioavailability of some nutrients [34]. Moreover, ingredients and compounds in UPF might contribute to metabolic derangements, negatively impacting adiposity and mitochondrial function [35].

In the last decades, a growing body of evidence has reported a positive association between UPF consumption and obesity, hypertension, diabetes, cancer, and mental health among adults [18,21,36,37,38,39]. Moreover, emerging research has suggested that higher UPF consumption is associated with poor maternal and neonatal outcomes [40,41], highlighting the possible effect of early exposure to undesirable compounds in UPF. In addition, a dose-response meta-analysis with seven cohort studies showed that for each 10% increase in UPF consumption as a contribution to daily energy intake, there was a 15% higher risk of all-cause mortality [42]. On the other hand, another meta-analysis with 40 prospective cohort studies explored the association between UPF groups and all-cause mortality and found that higher consumption of sugar-sweetened beverages, artificially sweetened beverages, processed meat, and red meat was positively associated with all-cause mortality, whereas breakfast cereals consumption was negatively associated with it [43]. It is noteworthy that UPF includes a wide range of products with different or similar nutritional compositions being its potential health impact distinct according to their food matrix or structures [44].

In the present study, when considering only UPF, more than one-quarter of dairy products and cereal, derivatives and tubers were labelled as Nutri-Score A. Additionally, yogurt and other fermented milk were the primary sub-group classified under Nutri-Score A. Romero Ferreiro et al. [25] reported similar findings, representing dairy products, ready meals, and canned dishes as the main UPF classified as Nutri-Score A. As the Nutri-Score only addresses food nutrient composition, it does not cover another dimension of foods, such as food processing. Therefore, two food products rated with the same letter and color by Nutri-Score may have different NOVA classifications [25]. In this study, for example, in the dairy products group, 15.3% of foods were classified with Nutri-Score A and NOVA 4, and only 1.1% were classified with Nutri-Score A and NOVA 1. Generally, food products rated as Nutri-Score A or B are perceived as healthier, increasing purchase intentions [45,46]. Thus, regardless of other individual determinants, if information on other dimensions of food is not provided, consumers may base their food choices mainly on the nutritional quality of food.

On the other hand, food manufacturers tend to reformulate their products to attain a better Nutri-Score by reducing the sugar, fat, and salt content or increasing the amount of fibre to capture consumers, regardless of the degree and extent of processing this reformulation requires [46]. A study carried out in France by Union Fédéral des Consommateurs (UFC-Que Choisir) showed that between 2015 and 2022, the nutritional quality improved in three of the seven food groups analyzed and in which Nutri-Score is more frequently displayed, namely in cereal bars, special breads and rusk, and breakfast cereals. For example, the proportion of highest nutritional quality categories (A and B) increased from 0% to 13% for cereal bars, 8% to 38% for breakfast cereals and 40% to 62% for special breads and rusk. Conversely, in the other four food groups analyzed (biscuits and cakes, bars and chocolate snacks, condiments sauces, ice cream and sorbets), the nutritional quality has not significantly improved. It is rarely displayed on the packaging [47].

In the present study, as expected, we found that the Nutri-Score positively correlates with the Multiple Traffic Lights system for total fat, saturated fat, total sugar, and salt, unveiling a possible clusterization between both FOPL systems. Moreover, although Nutri-Score does not consider food processing, we found a weak positive correlation between Nutri-Score and NOVA classification. Likewise, a meta-analysis including representative sample surveys from different countries found that increased consumption of UPF negatively affects the nutritional quality of diets, particularly through increasing intake of free sugars, total fats, and saturated fats and decreasing dietary protein sources [48].

As food processing and nutritional quality are two distinct dimensions of food, they may have an isolated or combined impact on the risk of chronic diseases. Many epidemiological studies have explored the isolated effect of food processing and nutritional quality of diet; however, few studies have explored their combined impact on health. The Moli-sani prospective cohort study analyzed the individual and combined association on diet quality, measured using the FSAm-NPS dietary index (underpinning the Nutri-Score) and UPF consumption (NOVA classification) to understand which dimension plays a major role in mortality in a large sample of Italian adults [49]. Their results indicated that adults with poor diets and higher consumption of ultra-processed food were at the highest risk of all-cause and cardiovascular disease mortality. When analyzing the combined association of these two dimensions, a significant attenuation of the association between the nutritional quality of the diet and all-cause and cardiovascular disease mortality was observed.

On the other hand, the effect of UPF intake remained largely unaltered for mortality risk. Similarly, a review of prospective cohort studies showed that the magnitude of the association between UPF consumption and obesity and health-related outcomes remained unchanged, even after adjustment for diet quality or pattern [50]. These findings suggest that UPF may negatively impact health, regardless of its nutritional value.

The contribution of ultra-processing and nutritional food quality to diet quality may also differ when considering their isolated or combined effects. The Nutri Net-Santé cohort study involving 98,454 French adults reported that the contribution to the total impact on overall diet quality was 26% for the nutritional quality of the foods consumed, 30% for UPF consumption and 44% for the combined nutritional quality of the foods consumed and UPF consumption [51]. Thus, since food nutritional quality and processing are not mutually exclusive, both should be considered as underpinning dimensions of the diet. Acknowledging this concern, it has been suggested that the degree of food processing and nutritional quality be incorporated into a single algorithm; however, evidence has shown that it is not feasible to generate an algorithm that encompasses both or all health dimensions of foods. Therefore, the approach to this issue should be geared towards labelling that considers the degree of food processing, such as a stop sign warning used in Chile or meaningful colors used in the Open Food Facts database for NOVA classification.

Some limitations of the present study should be acknowledged. It should be noted that mandatory information on food labelling needed to determine Nutri-Score was frequently missing for dietary fibre and the percentage of fruit and vegetables, nuts, and pulses. However, missing data were estimated according to previous procedures used in other studies [28,29]. The ability to discriminate food processing degree with food labelling information may lead to overestimation or underestimation of foods into different NOVA groups. Nonetheless, in addition to each NOVA group’s definition, examples of foods were identified. In addition, we cannot ignore the bias associated with selecting food products since most of the products chosen were processed and ultra-processed food products available in the Portuguese market. All queries were discussed and resolved in consensus among all authors.

## 5. Conclusions

Our findings indicate a large number of UPFs in all Nutri-Score categories. As food processing and nutritional quality are complementary, both should be considered in labelling. Food cannot be considered merely as a sum of nutrients, so government strategies should address the regulation of the ultra-processing of foods and improve the labelling and information available to the consumer.

## Figures and Tables

**Table 1:** Classification of products according to the amount of total fat, saturated fat, sugar and salt per 100 g or 100 mL [10].

	Total Fat	Saturated Fat	Sugar	Salt
**Foods**	g/100 g			
Low	≤3	≤1.5	≤5	≤0.3
Medium	3–17.5	1.5–5	5–22.5	0.3–1.5
High	>17.5	>5	>22.5	>1.5
**Beverages**	g/100 mL			
Low	≤1.5	≤0.75	≤2.5	≤0.3
Medium	1.5–8.75	0.75–2.5	2.5–11.25	0.3–0.75
High	>8.75	>2.5	>11.25	0.75

**Table 2:** Nutri-Score and NOVA classification distribution within food groups and subgroups (*n* = 2682).

		NOVA Classification				Nutri-Score					
Food Group	Food Subgroup	1	2	3	4	A	B	C	D	E	FSAm-NPS Score
		*n* (%)				*n* (%)					Median (P25; P75)
Fruits, vegetables and pulses (*n* = 146)	Vegetables (*n* = 31)	8 (25.8)	0 (0.0)	3 (9.7)	20 (64.5)	20 (62.5)	5 (16.1)	5 (16.1)	1 (3.2)	0 (0.0)	−2.0 (−6.0; 1.0)
	Nuts and seeds (*n* = 30)	19 (63.3)	0 (0.0)	9 (30.0)	2 (6.7)	9 (30.0)	10 (33.3)	6 (20.0)	5 (16.7)	0 (0.0)	2.0 (−1.0; 5.5)
	Processed fruit (*n* = 33)	14 (42.4)	0 (0.0)	13 (39.4)	6 (18.2)	6 (18.2)	11 (33.3)	14 (42.4)	2 (6.1)	0 (0.0)	2.0 (0.0; 4.0)
	Pulses (*n* = 52)	3 (5.8)	0 (0.0)	35 (67.3)	14 (26.9)	44 (84.6)	3 (5.8)	5 (9.6)	0 (0.0)	0 (0.0)	−8.0 (−8.0; −4.0)
Dairy products (*n* = 452)	Milk (*n* = 43)	0 (0.0)	0 (0.0)	2 (4.7)	41 (95.3)	16 (37.2)	24 (55.8)	1 (2.3)	1 (2.3)	1 (2.3)	0.0 (−1.0; 1.0)
	Dairy cream (*n* = 17)	0 (0.0)	1 (5.9)	0 (0.0)	16 (94.1)	0 (0.0)	0 (0.0)	0 (0.0)	17 (100)	0 (0.0)	14.0 (12.5; 14.5)
	Yogurt and other fermented milk (*n* = 244)	12 (4.9)	0 (0.0)	9 (3.7)	223 (91.4)	58 (23.8)	124 (50.8)	61 (25.0)	1 (0.4)	0 (0.0)	1.0 (0.0; 3.0)
	Cheese and Curd (*n* = 148)	0 (0.0)	0 (0.0)	45 (30.4)	103 (69.6)	0 (0.0)	15 (10.1)	37 (25.0)	46 (31.1)	50 (33.8)	6.5 (4.0; 11.0)
Cereals, derivatives and tubers (*n* = 324)	Rice and other grains (*n* = 15)	3 (20.0)	0 (0.0)	7 (46.7)	5 (33.3)	8 (53.3)	4 (26.7)	3 (20.0)	0 (0.0)	0 (0.0)	−1.0 (−4.0; 2.0)
	Potatoes and other tubers (*n* = 5)	0 (0.0)	0 (0.0)	2 (40.0)	3 (60.0)	2 (40.0)	2 (40.0)	1 (20.0)	0 (0.0)	0 (0.0)	0.0 (−2.5; 3.5)
	Bread and toast (*n* = 96)	0 (0.0)	0 (0.0)	0 (0.0)	96 (100)	36 (37.5)	26 (27.1)	23 (24.0)	11 (11.5)	0 (0.0)	0.0 (−2.5; 3.5)
	Flours, pasta for bread and pastries (*n* = 14)	2 (14.3)	0 (0.0)	0 (0.0)	12 (85.7)	6 (42.9)	1 (7.1)	6 (42.9)	1 (7.1)	0 (0.0)	2.0 (−4.0: 8.0)
	Breakfast cereals and cereal bars (*n* = 194)	7 (3.6)	0 (0.0)	1 (0.5)	186 (95.9)	34 (17.5)	20 (10.3)	72 (37.1)	62 (32.0)	6 (3.1)	9.0 (0.0; 12.0)
Meat, seafood and eggs (*n* = 459)	Meat (*n* = 381)	1 (0.3)	0 (0.0)	16 (4.2)	364 (95.5)	2 (0.5)	24 (6.3)	38 (10.0)	147 (38.6)	170 (44.6)	17.0 (11.0; 23.0)
	Seafood (*n* = 78)	0 (0.0)	0 (0.0)	35 (44.9)	43 (55.1)	8 (10.3)	46 (59.0)	21 (26.9)	3 (3.8)	0 (0.0)	2.0 (0.0; 3.0)
Oils and fats (*n* = 101)	Vegetal oils (*n* = 22)	0 (0.0)	18 (81.8)	0 (0.0)	4 (18.2)	0 (0.0)	0 (0.0)	1 (4.5)	17 (77.3)	4 (18.2)	11.0 (11.0; 13.0)
	Olive oil (*n* = 16)	0 (0.0)	13 (81.3)	0 (0.0)	3 (18.8)	0 (0.0)	0 (0.0)	16 (100.0)	0 (0.0)	0 (0.0)	6.0 (6.0; 6.0)
	Butter (*n* = 40)	0 (0.0)	7 (17.5)	21 (52.5)	12 (30.0)	0 (0.0)	0 (0.0)	0 (0.0)	6 (15.0)	34 (85.0)	23.0 (19.0; 25.0)
	Margarines and minarines (*n* = 18)	0 (0.0)	0 (0.0)	0 (0.0)	18 (100)	0 (0.0)	0 (0.0)	6 (33.3)	8 (44.4)	4 (22.2)	14.0 (9.8; 17.5)
	Other fats (*n* = 5)	0 (0.0)	0 (0.0)	0 (0.0)	5 (100)	0 (0.0)	0 (0.0)	0 (0.0)	4 (80.0)	1 (20.0)	16.0 (13.0; 19.0)
Sweets, cakes and cookies (*n* = 576)	Sweets (*n* = 303)	0 (0.0)	0 (0.0)	4 (1.3)	299 (98.7)	6 (2.0)	7 (2.3)	71 (23.4)	101 (33.3)	118 (38.9)	16.0 (10.0; 22.0)
	Cakes (*n* = 37)	0 (0.0)	0 (0.0)	0 (0.0)	37 (100)	0 (0.0)	2 (5.4)	2 (5.4)	17 (45.9)	16 (43.2)	18.0 (14.5; 20.5)
	Biscuits and commercial cookies (*n* = 236)	0 (0.0)	0 (0.0)	0 (0.0)	236 (100)	3 (1.3)	6 (2.5)	47 (19.9)	89 (37.7)	91 (38.6)	17.0 (11.0; 22.0)
Snacks, pretzels and pizzas (*n* = 264)	Snacks and packed chips (*n* = 167)	0 (0.0)	0 (0.0)	66 (39.5)	101 (60.5)	5 (3.0)	9 (5.4)	78 (46.7)	59 (35.3)	16 (9.6)	10.0 (8.0; 14.0)
	Stuffed and fried patties and pizzas (*n* = 97)	0 (0.0)	0 (0.0)	0 (0.0)	97 (100)	6 (6.2)	30 (30.9)	37 (38.1)	23 (23.7)	1 (1.0)	4.0 (2.0; 10.5)
Meat substitutes (*n* = 58)		0 (0.0)	0 (0.0)	2 (3.4)	56 (96.6)	25 (43.1)	9 (15.5)	14 (24.1)	9 (15.5)	1 (1.7)	0.5 (−2.0; 9.3)
Milk and dairy products substitutes (*n* = 65)		2 (3.1)	0 (0.0)	5 (7.7)	58 (89.2)	17 (26.2)	37 (56.9)	1 (1.5)	2 (3.1)	8 (12.3)	0.0 (−1.0; 1.0)
Ready meals (*n* = 35)		0 (0.0)	0 (0.0)	4 (11.4)	31 (88.6)	6 (17.1)	13 (37.1)	13 (37.1)	3 (8.6)	0 (0.0)	2.0 (0.0; 5.0)
Others (*n* = 54)		0 (0.0)	0 (0.0)	0 (0.0)	54 (100.0)	2 (3.7)	0 (0.0)	27 (50.0)	17 (31.5)	8 (14.8)	10.0 (6.0; 16.0)
Non-alcoholic beverages (*n* = 148)	Natural fruit juices and 100% juices (*n* = 23)	9 (39.1)	0 (0.0)	9 (39.1)	5 (21.7)	0 (0.0)	3 (13.0)	10 (43.5)	6 (26.1)	4 (17.4)	5.0 (3.0; 7.0)
	Nectars (*n* = 21)	0 (0.0)	0 (0.0)	0 (0.0)	21 (100.0)	0 (0.0)	0 (0.0)	4 (19.0)	2 (9.5)	15 (71.4)	11.0 (6.5; 13.0)
	Soft drinks (*n* = 104)	0 (0.0)	0 (0.0)	0 (0.0)	104 (100.0)	0 (0.0)	12 (11.5)	23 (22.1)	38 (36.5)	31 (29.8)	6.0 (4.0; 11.0)

## Data Availability

The data will be available upon reasonable request.

## Appendix A

**Table A1:** Groups and subgroups classification according to FoodEx 2 [26].

Groups and Subgroups		Foods Included
Fruits, vegetables and pulses		
Vegetables	Fresh	Vegetables in natura.
	Processed	Frozen and canned vegetables, pickles and pates.
Nuts and seeds	Nuts	Various nuts, including almonds, peanuts, walnut and cashew.
	Seeds	Various seeds, including flaxseed, chia, pumpkin and pine nuts.
	Processed nuts	Caramelized nuts, peanut and almond butter, tahini.
Fresh fruit	Fresh fruit	Fruit in natura
	Fruit jars	Commercial fruit jars intended for infant feeding
Processed fruit	Canned fruit	Canned fruit in sugar syrup.
	Dehydrated fruit	Dried and dehydrated fruit.
Pulses		Dried and fresh pulses, including beans, chickpeas, green peas, broad beans and lentils.
Soup		
Soups		Vegetable, meat and fish soups
Dairy products		
Milk	Milk	Cow’s milk, goat and sheep milk, fat milk, half-fat and skimmed milk, lactose-free milk, and easy-to-digest milk.
	Processed milk	Milkshakes, chocolate milk and flavoured milk.
	Milk powder, condensed and evaporated	Milk powder, condensed milk and evaporated milk.
Dairy cream		Pasteurized and UHT cream, whipped cream and flavoured cooking cream.
Yogurt and other fermented milk		Solid yogurts and fermented solid milk, liquid yogurts, fat and skimmed yogurts and kefir.
Cheese and Curd		Goat, cow, sheep, fresh, cured, cream and curd cheese and protected designation of origin (PDO) products.
Cereals, derivatives and tubers		
Pasta		Fresh and dry pasta, stuffed pasta, whole and gluten-free pasta.
Rice and other grains	Rice	Common rice, brown and wild rice.
	Other grains	Various grains, including corn, buckwheat, quinoa, bulgur and oats.
Potatoes and other tubers		Potato, sweet potato, yam, cassava.
Bread and toasts		All kinds of bread of different cereals, including bread, toast, bread, breadcrumbs, and gressinos.
Flour, pasta for bread and pastries		Flours, starches, flakes, semolinas, pasta for bread, pizza dough, broken dough, puff pastry and sanded.
Infant cereals		Dairy and non-dairy flour
Breakfast cereals and cereal bars	Breakfast cereals	Sugary cereals, muesli, granola, bran.
	Cereal bars	Sugary cereal bars, simple, with fruit, with chocolate.
Meat, seafood and eggs		
Meat	Poultry meat and breeding	Chicken meat, turkey, rabbit, hare, pigeon, quail, duck
	Red meat	Beef, veal, goat, lamb, lamb, pork, boar, horse, goat.
	Entrails	Various entrails include chicken, pig, cow, veal, and sheep.
	Cold cuts and other processed meats	Cold cuts and other meats
Seafood	Fresh, dry and canned fish	Fresh, dried, canned fish, fish roe, and dried fish, including codfish and smoked salmon.
	Crustaceans, molluscs, derivatives and other	Octopus, squid, shrimp, clams, mussels, oysters, including canned.
	Processed fish	Fish fingers, whims of the sea, pates, surimi, fish pastes.
Eggs		Chicken eggs, quail, egg powder, liquid egg, egg white.
Oils and fats		
Vegetal oils		Peanut oil, palm, soybean, corn, sunflower and mixtures.
Olive oil		Olive oil
Butter		Salted butter, unsalted butter, lactose-free butter.
Margarines and minarines		Vegetable creams, minarines, margarines, industrial fats
Other fats		Fish oil, lard and sebum
Sweets, cakes and cookies		
Sweets	Added sugar	White sugar, brown, demerara, vanilla.
	Honey, molasses and syrup	Honey, molasses and syrup
	Jellies, jams and candied fruits	Jams, fruit jam, jellies, marmalade, guava jelly, candied fruits.
	Sweets, gums and chewing gum	Sweets, jellybeans and gums.
	Chocolates and chocolate snacks	Chocolates and chocolate snacks.
	Ice cream	Milk and cream ice creams and sorbets
	Sweet desserts	Dairy desserts, chocolate mousse, fruit mousses, eggs-based Portuguese desserts and egg creams, gelatine.
Cakes		Cakes, pies, croissants and other pastries with or without cream, including homemade recipes.
Biscuits and commercial cookies		Cookies, water and salt crackers, chocolate and stuffed cookies, cookies with topping, whole cookies and other types.
Artificial sweeteners		
Artificial sweeteners		Aspartame, sucrose, sucralose, stevia, sodium cyclamate.
Snacks, pretzels and pizzas		
Snacks and packed chips		Bread snacks, packed chips, salted popcorn and packaged fried snacks.
Stuffed and fried patties and pizzas		Patties, croquettes, codfish cakes, pies, meatballs, puff pastry and pizzas.
Meat substitutes		
Meat substitutes		Vegetable burger, vegetable sausage, tofu, seitan, veggie pâté.
Milk and dairy products substitutes		
Milk and dairy products substitutes		Coconut, oat and soy drinks, soy yogurt, vegetable yogurt, soy dessert, non-dairy cream.
Adding salt		
Adding salt		Coarse salt, table salt, iodate salt and salt flower.
Others		
Others		Yeasts and gelatines, aromas and essentials, herbs and spices, condiments, sauces and mayonnaises, broths and soups powdered.
Non-alcoholic beverages		
Water		Natural mineral water, carbonated mineral water, and flavoured water.
Tea and infusions		Black and green tea, herbs and fruit infusions.
Coffee		Coffee, decaffeinated, coffee mixes, chicory, substitutes and coffee substitutes.
Natural fruit juices and 100% juices		Natural fruit juices, 100% fruit and vegetable juices
Nectars		Fruit and vegetable nectars, light nectars.
Soft drinks		Soft drinks with and without gas, lemonade, tonic water, energy drinks, concentrated juices.
Other non-alcoholic beverages		Isotonic drinks, non-alcoholic beer and non-alcoholic cocktails.
Alcoholic beverages		
Wine		White, green and red wine.
Generous wines and liqueurs		Porto wine, *moscatel*, liqueurs, Martini.
Beer		White, black and redhead beer
Distilled beverages		Whisky, brandy, tequila, rum.
Other alcoholic beverages		Cider, *sangria*, *panaché*, *poncha.*
